# Antagonistic Effect of Azoxystrobin Poly (Lactic Acid) Microspheres with Controllable Particle Size on *Colletotrichum higginsianum* Sacc

**DOI:** 10.3390/nano8100857

**Published:** 2018-10-19

**Authors:** Junwei Yao, Bo Cui, Xiang Zhao, Heng Zhi, Zhanghua Zeng, Yan Wang, Changjiao Sun, Guoqiang Liu, Jinming Gao, Haixin Cui

**Affiliations:** 1 Institute of Environment and Sustainable Development in Agriculture, Chinese Academy of Agricultural Sciences, Beijing 100081, China; yaojunwei@nwafu.edu.cn (J.Y.); cuibo@caas.cn (B.C.); zhaoxiang@caas.cn (X.Z.); zhiheng6996@163.com (H.Z.); zengzhanghua@caas.cn (Z.Z.); wangyan03@caas.cn (Y.W.); sunchangjiao@caas.cn (C.S.); liuguoqiang@caas.cn (G.L.); 2 Shaanxi Key Laboratory of Natural Products & Chemical Biology, College of Chemistry & Pharmacy, Northwest A&F University, Yangling 712100, China

**Keywords:** azoxystrobin, microsphere, *Colletotrichum higginsianum* Sacc, oxidative damage, antagonistic activity

## Abstract

Size-controlled azoxystrobin-poly (lactic acid) microspheres (MS) were prepared by an oil/water emulsion solvent evaporation approach. The hydrated mean particle sizes of the MS1, MS2, and MS3 aqueous dispersions were 130.9 nm, 353.4 nm, and 3078.0 nm, respectively. The drug loading and encapsulation efficiency of the azoxystrobin microspheres had a positive relationship with particle size. However, the release rate and percentage of cumulative release were inversely related to particle size. The smaller-sized microspheres had a greater potential to access the target mitochondria. As a result, the more severe oxidative damage of *Colletotrichum higginsianum* Sacc and higher antagonistic activity were induced by the smaller particle size of azoxystrobin microspheres. The 50% lethal concentrations against *Colletotrichum higginsianum* Sacc of MS1, MS2, and MS3 were 2.0386 μg/mL, 12.7246 μg/mL, and 21.2905 μg/mL, respectively. These findings reveal that particle size is a critical factor in increasing the bioavailability of insoluble fungicide.

## 1. Introduction

Pesticides, especially insecticides, fungicides, and herbicides, play a critical role in controlling crop disasters and maintaining the steady growth of agricultural yields [1]. However, the low water solubility of pesticide compounds can inhibit their applications. For conventional pesticide formulations, poor dispersibility, droplet drift, and photolysis result in low bioavailability and environmental pollution [2]. Based on the Nernst–Brunner equation, the dissolution rate of an insoluble ingredient is increased with the reduction of particle size. Recently, constructing nanoformulations with small particle size and large surface areas has attracted significant attention, particularly in the agricultural field [3,4]. 

**Figure 1:**
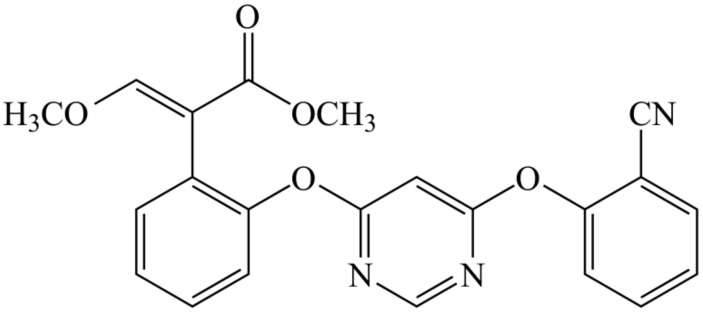
Molecular structure of azoxystrobin.

*Brassica rapa pekinensis* is a significant component of the human diet, providing essential vitamins, minerals, and dietary fibers [5,6,7]. Like other vegetables, *Brassica rapa pekinensis* is subjected to damage caused by diseases, such as *Colletotrichum higginsianum* Sacc [8]. In order to guarantee the yield and quality of Brassica rapa pekinensis, fungicides are widely used in its cultivation. Strobilurins are natural substances primarily derived from Oudemansiella mucida and Strobilurus tenacellus. Strobilurins bind to a single site in the inner mitochondrial membrane, the quinol oxidation (Qo) site of cytochrome bc1 enzyme complex III [9,10]. Hence, the strobilurins are identified as Qo inhibitors (QoI). QoI block electron transport from cytochrome b to cytochrome c1, which leads to a lack of metabolic energy supply by reducing oxidation of reduced nicotinamide adenine dinucleotide (NADH) and synthesis of adenosine triphosphate (ATP) [11,12]. The mechanism of strobilurins is novel and targeted. The most important features of strobilurin fungicides are high activity and rapid effect. Azoxystrobin (AZ), [methyl(E)-2-{2-[6-(2-cyanophenoxy)-pyrimidin-4-yloxy]phenyl}-3-methoxyacrylate] (see Figure 1), is a broad-spectrum of QoI strobilurin fungicides. However, the low solubility of azoxystrobin in aqueous solution (6.7 μg/mL) severely restricts its efficient application in crop protection [13]. The improper and excessive use of azoxystrobin could result in QoI resistance and injuries to non-target species, affecting species diversity and the ecosystem. Therefore, improving azoxystrobin bioavailability has a great scientific and practical research value. 

The biocompatible polymers of poly(lactic acid) (PLA) and poly(lactide-co-glycolide) (PLGA), approved by the US Food and Drug Administration (FDA), have become a joint focus of attention in the field of nanomedicine scientific research, because the polymers show good biodegradability and are known as the carriers of a drug delivery system [14,15]. In recent years, PLA and PLGA have been widely used as carrier material for drugs with a short half-life, poor stability, easy degradation and severe toxicities to control drug release [16,17,18]. The molecular weight and the dosage of PLA could affect the drug loading and encapsulation efficiency of microspheres. In addition, the stable and efficient release of active ingredients are achieved by controlling the ratio of drugs to PLA. Hydrophobic PLA carriers have been widely used in the preparation of microspheres and microcapsules with a high drug loading rate, enhanced bioavailability, reduced side effects, and biological degradability [19,20]. The application of nanoscience and nanotechnology in the development of pesticide formulations has provided dominant potential to increase the pesticides efficiency and improve the ecological environment [21]. In this case, the novel pesticide formulation produced by nanotechnology has emerged as a potential revolution in agricultural production [22,23,24]. It is known that smaller-size particles can possess better permeability and larger surface/volume ratio to achieve a better control effect [25]. Therefore, it is important to reveal particle size-dependent effects on drug loading, release rate and control release.

In this study, the size-controlled azoxystrobin-loaded PLA microspheres were prepared by an oil/water (*o/w*) emulsion solvent evaporation method. The hydrated mean particle size and polydispersity index (PDI) of microspheres were measured by dynamic light scattering (DLS). The morphology of microspheres was observed by transmission electron microscopy (TEM). The drug loading and encapsulation efficiency of azoxystrobin in lyophilized microspheres were evaluated by high-performance liquid chromatography (HPLC) quantitative analysis. The release behavior of azoxystrobin microspheres was examined in vitro. The contact angles and storage stability of microspheres were also measured. The antagonistic activities of azoxystrobin microspheres were performed in a potato dextrose agar (PDA) Petri dish against *Colletotrichum higginsianum* Sacc. To illustrate the particle size effect on the antifungal efficacy, the contents of reactive oxygen species (ROS) and antioxidase activities were determined to reveal the oxidative damage caused by azoxystrobin microspheres with different particle sizes.

## 2. Experimental Section

### 2.1. Materials

Azoxystrobin (97%) was obtained from Hubei Sheng Tianheng record Biological Technology Co., Ltd. (Wuhan, China). PLA (Mw ~ 100 kDa) was supplied by Daigang Biomaterial Co., Ltd. (Jinan, China). Coumarin 6 and poly(vinyl alcohol) (PVA), with a degree of polymerization of 2000 and a degree of hydrolysis of 86% were purchased from J&K Scientific Ltd. (Beijing, China). Dichloromethane (DCM) was purchased from Sinopharm Chemical Reagent Co., Ltd. (Shanghai, China). HPLC-grade methanol (MeOH) was purchased from Thermo Fisher Scientific (Shanghai, China). Water purified by Milli-Q system (18.2 MΩ cm, TOC ≤ 4 ppb, Dubuque, IA, USA) was used for all experiments.

### 2.2. Methods

#### 2.2.1. Preparation of Azoxystrobin Microspheres

Azoxystrobin microspheres were prepared by an *o/w* solvent evaporation approach. Briefly, 160 mg of PLA, 1 mg of coumarin 6 and 40 mg of azoxystrobin powder were dissolved in 8 mL of DCM to form an organic phase. The aqueous phase was composed of 0.6% (*w*/*v*) PVA as an emulsifier. The 8 mL of organic solution was dropped into 40 mL of aqueous solution via magnetic stirring. Then, the mixed solution was emulsified using probe ultrasonication (JY92-IIN, Scientz, Ningbo, China) at 390 W (interval/pause = 20:10 s) in an ice bath for 4 min. The coarse emulsion was penetrated through Shirasu Porous Glass (SPG) hydrophilic membrane (KT-125, SPG Technology, Miyazaki, Japan) with a uniform pore size of 0.1 μm and 0.3 μm into the external water phase under 0.2 MPa pressure of nitrogen gas, to get the microsphere 1 (MS1) and microsphere 2 (MS2) final emulsions, respectively. To obtain the microsphere 3 (MS3) final emulsion, after dropping the organic solution into the aqueous phase, the mixed solution was emulsified in an ultrasonic cleaner (KQ-500DE, Kun Shan Ultrasonic Instruments, Suzhou, China) at 80% power for 5 min. Then, the above three final emulsions were solidified by magnetic stirring for 4 h at room temperature (RT). The solidified solution was then evaporated by a rotavapor (RE100-Pro, SCILOGEX, Rocky Hill, CT, USA) to remove the residual solvent of DCM. The coumarin 6-loaded azoxystrobin microspheres were collected by centrifugation (Sorvall ST 16 R, Thermo Fisher Scientific, Pittsburgh, PA, USA) at 10,000 rpm and 16 °C for 20 min, followed by washing with deionized water three times. Finally, the water from the azoxystrobin microspheres was eliminated by lyophilizer (FD-81, EYELA, Tokyo, Japan) after liquid nitrogen freezing for 48 h. Non-coumarin 6-loaded azoxystrobin microspheres were fabricated by following the same processes except that coumarin 6 was not added into the organic phase.

#### 2.2.2. Size and Morphological Characterizations of Microspheres

The hydrated particle sizes, PDIs, and zeta potentials of the aqueous diluted azoxystrobin PLA microspheres (500 μg/mL) were measured using a Zetasizer (Nano ZS90, Malvern Instruments, Worcestershire, UK) at RT in triplicate. In the morphological characterization, 3 μL diluted dispersion of the microspheres (50 μg/mL) was placed onto a carbon film supported on a 300-mesh copper grid. The morphologies of the microspheres were observed by a TEM (HT7700, Hitachi, Tokyo, Japan) at 80 kV of accelerating voltage. The dried particle size distribution of microspheres was calculated using Nano Measurer 1.2.5 software (Fudan University, Shanghai, China) by measuring 100 particles based on the TEM images.

#### 2.2.3. Drug Loading and Encapsulation Efficiency of Microspheres

The drug loading and encapsulation efficiency of azoxystrobin in lyophilized microspheres were evaluated by dissolving the microspheres completely in DCM. After removing DCM via reduction vaporization, the dried microspheres were dispersed in MeOH with vigorous vortex mixing and sonication at RT. Then, the azoxystrobin solutions were filtered by a 0.22 μm membrane. The quantitative analysis of azoxystrobin was conducted by HPLC (1260 series, Agilent Technologies, Palo Alto, CA, USA) using a C_18_ analysis column (150 mm × 4.6 mm × 5 μm) with a UV detector at 254 nm. The mobile phase was MeOH/H_2_O (75:25, *v/v*). The flow rate was 0.8 mL/min. Each independent experiment was repeated three times.

$$\mathrm{Drug}\;\mathrm{loading}\;\%\;=\;\frac{\mathrm{weight}\;\mathrm{of}\;\mathrm{azoxystrobin}\;\mathrm{in}\;\mathrm{lyophilized}\;\mathrm{micropheres}}{\mathrm{weight}\;\mathrm{of}\;\mathrm{lyophilized}\;\mathrm{micropheres}}\times 100$$

$$\mathrm{Encapsulation}\;\mathrm{efficiency}\;\%\;=\;\frac{\mathrm{weight}\;\mathrm{of}\;\mathrm{azoxystrobin}\;\mathrm{in}\;\mathrm{lyophilized}\;\mathrm{micropheres}}{\mathrm{weight}\;\mathrm{of}\;\mathrm{input}\;\mathrm{azoxystrobin}}\times 100$$

#### 2.2.4. In Vitro Release of Azoxystrobin from Microspheres

The azoxystrobin release curve profiles were estimated in MeOH/H_2_O (50:50, *v/v*) release medium [26,27]. The lyophilized microspheres with equivalent active ingredient were put in a dialysis bag (MWCO 2000 Da) containing 3 mL of release medium. After that, the dialysis bag was immersed in 97 mL of the release medium. The release system was shaken at 100 rpm at 28 °C using a thermostatic incubator (THZ-98C, Yiheng Scientific, Shanghai, China). At different timed intervals, 2 mL of the outside dialysis solution was withdrawn and analyzed by HPLC. The same volume of fresh medium was supplemented into the release system after each sampling. All experiments were conducted independently three times.

#### 2.2.5. Stability Evaluation of MS1 Microspheres

The physicochemical stability of the azoxystrobin MS1 was evaluated at three different temperatures. The MS1 samples were placed in glass vials and three parallel tests were set up. After storage at 4 °C, 25 °C, and 54 °C for 14 d, the variation in hydrated particle size was estimated by DLS and the active ingredient in MS1 was analyzed by HPLC.

#### 2.2.6. Contact Angle Analysis of Microspheres

Fresh cucumber and cabbage leaves were cultivated by manual climatic incubator (430D, Ningbo Jiangnan Instrument, Ningbo, China). The static contact angles of the azoxystrobin microspheres (400 μg/mL) were determined by an angle apparatus (JC2000D2M, Zhongchen Digital Technology Apparatus, Shanghai, China). The 5 μL diluted solution was added to the surface of the fresh leaves. Each experiment was independently performed five times at RT.

#### 2.2.7. In Vitro Antagonistic Activity Assay

To evaluate the fungal antagonistic activity of azoxystrobin microspheres with different particle sizes, *Colletotrichum higginsianum* Sacc was used as the model for the fungal disease in vitro test. The formulations of azoxystrobin MS1, MS2, or MS3 were accurately diluted with sterile melted PDA medium to the final concentrations: 0.25 μg/mL, 0.5 μg/mL, 1.0 μg/mL, 5.0 μg/mL, and 10.0 μg/mL. Equal volumes of sterile deionized water were added into the sterile melted PDA medium as the control check (CK) group. *Colletotrichum higginsianum* Sacc was inoculated into the PDA at 27 ± 1 °C in the dark for four days. Subsequently, a 5 mm-diameter mycelial disk was punched from the actively growing colonies and placed in the center of the tested PDA plate having a diameter of 90 mm. The fungal antagonistic assay was independently conducted three times. After incubating at 27 °C in the dark for two days, the colony diameter of mycelium was measured by the criss-cross method. The percentage of the tested *Colletotrichum higginsianum* Sacc growth inhibition was evaluated by the following formula:

$$I\;\%\;=\;\frac{D_{c}-D_{t}}{D_{c}-D_{d}}\times 100$$

where I% is the inhibition ratio of the tested *Colletotrichum higginsianum* Sacc growth. D_c_ and D_t_ are the *Colletotrichum higginsianum* Sacc growth diameter in the control check group and azoxystrobin-treated groups, respectively. D_d_ is the diameter of the mycelial disk (5 mm). The toxicity regression equations of various azoxystrobin formulations were determined by probit analysis using IBM SPSS 20.0 software (IBM Corp., Armonk, NY, USA), following the medial lethal concentration (LC_50_).

#### 2.2.8. Mycelial Uptake Efficiency Assay

In the morphologically fluorescent characterization, 5 μL diluted dispersion of coumarin 6-loaded azoxystrobin microspheres (15 μg/mL) was placed onto a glass slide. After natural drying at RT, the green fluorescence microscopy images of coumarin 6-loaded azoxystrobin microspheres were observed by an inverted fluorescence microscope (IX71, Olympus Corp., Tokyo, Japan). The mycelial intracellular uptake efficiencies of azoxystrobin microspheres were determined by the fluorescent intensity of coumarin 6. In short, *Colletotrichum higginsianum* Sacc was inoculated into the PDA at 27 ± 1 °C in the dark for four days. A 5-mm-diameter mycelial disk was punched from the actively four-day growing colonies and inoculated into the potato dextrose broth (PDB) in the dark for two days. The coumarin 6-loaded MS1, MS2, or MS3 with final concentration of 15 μg/mL azoxystrobin was added to the above PDB inoculated with *Colletotrichum higginsianum* Sacc for one day. Then, the mycelia of *Colletotrichum higginsianum* Sacc were washed with phosphate buffered saline (PBS, 10 mM, pH 7.4) three times. Finally, the fresh weights of *Colletotrichum higginsianum* Sacc were measured and the mycelia were suspended in 2 mL PBS. The fluorescence intensity of mycelial intracellular coumarin 6-loaded azoxystrobin microspheres was measured by a fluorescent spectrophotometer (F-4600, Hitachi Corp., Tokyo, Japan) at 430 nm excitation and 502 nm emission, for coumarin 6. All intracellular uptake experiments were independently repeated five times.

#### 2.2.9. Reactive Oxygen Species Assay

Intracellular ROS level was determined by commercial ROS assay kit (E004, Nanjing Jiancheng Bioengineering Institute, Nanjing, China) using 2’,7’-dichlorofluorescin diacetate (DCFH-DA) as fluorescent probe. In brief, *Colletotrichum higginsianum* Sacc was inoculated into the PDA at 27 ± 1 °C in the dark for four days. A 5-mm-diameter mycelial disk was punched from the actively four-day growing colonies and inoculated into the PDB in the dark for two days. The MS1, MS2, or MS3 with final concentration of 15 μg/mL azoxystrobin was added to the above PDB inoculated with *Colletotrichum higginsianum* Sacc for two days, and an equal volume of sterile deionized water was used for the CK sample. Then, the final concentration of DCFH-DA (10 μM) was inoculated with *Colletotrichum higginsianum* Sacc in the PDB medium for 2 h. The mycelia of *Colletotrichum higginsianum* Sacc were washed with PBS (10 mM, pH 7.4) twice. Finally, the fresh weights of *Colletotrichum higginsianum* Sacc were measured and the mycelia were suspended in 2 mL PBS. The bright field and green fluorescent microscopy images of mycelia were performed by an inverted fluorescence microscope (IX71, Olympus Corp., Tokyo, Japan). The fluorescence intensity of *Colletotrichum higginsianum* Sacc was examined by a fluorescent spectrophotometer (F-4600, Hitachi Corp., Tokyo, Japan) at 480 nm excitation and 526 nm emission. All independent experiments were repeated five times.

#### 2.2.10. Oxidative Stress Assay

A 5-mm-diameter *Colletotrichum higginsianum* Sacc disk was inoculated into the PDB in the dark for two days. The mycelia were exposed to the PDB media of MS1, MS2, or MS3 containing 15 μg/mL azoxystrobin for two days, with an equal amount of sterile deionized water as CK. The fresh weights of mycelia were measured. The mycelia were homogenized in pre-cooled PBS with an automatic tissue grinder (Tissuelyser-24, Shanghai Jingxin Corp., Shanghai, China). The homogenate was centrifuged at 10,000 rpm for 20 min at 4 °C (Sorvall ST 16R, Thermo Fisher Scientific, Pittsburgh, PA, USA) to collect the supernatant for analyzing the antioxidant enzyme activity. The soluble protein of *Colletotrichum higginsianum* Sacc was tested using the Bradford assay kit (A045-2, Nanjing Jiancheng Bioengineering Institute, Nanjing, China). The antioxidant enzyme activities of superoxide dismutase (SOD) and catalase (CAT) were measured by an UV spectrophotometer (UV-2600, Shimadzu Corp., Kyoto, Japan) with commercial assay kits (A001-2-1, A007-1-1, Nanjing Jiancheng Bioengineering Institute, Nanjing, China). Each experiment was independently repeated three times.

#### 2.2.11. Statistical Analysis

The data analysis was carried out using IBM SPSS 20.0 software (IBM Corp., Armonk, NY, USA). The statistical data were expressed as mean ± standard deviation (SD) and calculated by a one-way analysis of variance (ANOVA) followed by the Student–Newman–Keuls (SNK) test. The value of probability less than 0.05 indicated significant differences between the experimental groups.

## 3. Results and Discussion

### 3.1. Characterization of the Microspheres

**Figure 2:**
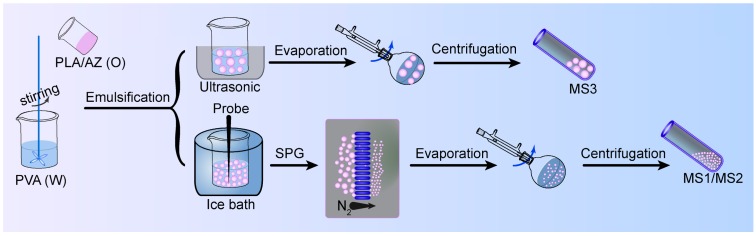
Schematic illustration of the preparation of poly(lactic acid) (PLA) microspheres (MSs).

**Figure 3:**
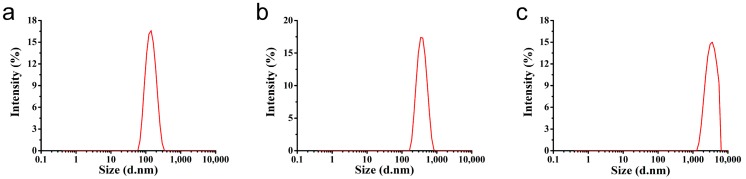
Hydrodynamic particle size of the azoxystrobin microspheres. (**a**) MS1; (**b**) MS2; (**c**) MS3.

As shown in Figure 2, the azoxystrobin-loaded PLA microspheres were produced by an *o/w* solvent evaporation method. Based on Figure 3, the hydrodynamic mean particle sizes of the aqueous diluted MS1, MS2, and MS3 determined by DLS were 130.9 ± 0.2 nm, 353.4 ± 6.3 nm and 3078.0 ± 336.6 nm, respectively. The PDI was set as a criterion of the particle size distribution [28,29]. The value of PDI less than 0.25 demonstrated a narrow distribution [30,31,32]. The PDIs of MS1 and MS2 aqueous dispersions were measured to be 0.10 ± 0.02 and 0.07 ± 0.05, showing monodispersity via SPG emulsification. As previously reported, the dexamethasone-poly[(d,l-lactide-co-glycolide)-co-poly ethylene glycol] (DEX-PLGA-PEG) nanoparticles were prepared by SPG membrane-assisted nanoprecipitation with PDI lower than 0.2 [33]. In addition, avermectin starch microparticles were fabricated by SPG membrane emulsification with PDI lower than 0.1 [34]. The PDI of the MS3 aqueous dispersion was 0.31 ± 0.02. This result indicated that the system treated by ultrasound showed wider distribution than the SPG method.

**Figure 4:**
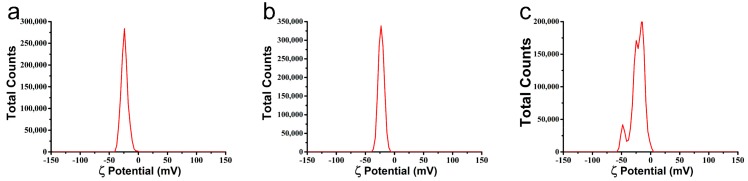
Zeta potentials of the azoxystrobin microspheres. (**a**) MS1; (**b**) MS2; (**c**) MS3.

The absolute value of zeta potential was a positive correlation with the stability of aqueous dispersion [35,36,37,38]. As shown in Figure 4, the zeta potentials of the MS1, MS2, and MS3 aqueous dispersions were −23.9 ± 0.3 mV, −22.4 ± 0.8 mV and −21.6 ± 1.7 mV, respectively. There was no significant difference between the three PLA microspheres. The negative zeta potentials were mainly derived from hydrolysis carboxyl groups of PLA and were independent of the particle sizes.

**Figure 5:**
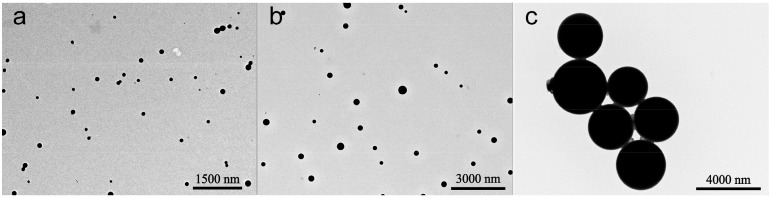
Transmission electron microscopy (TEM) morphologies of the azoxystrobin microspheres. (**a**) MS1; (**b**) MS2; (**c**) MS3.

**Figure 6:**
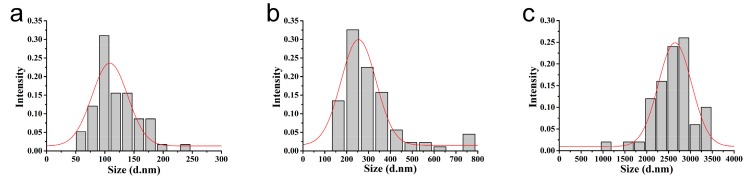
Statistical dried particle sizes distributions of azoxystrobin microspheres based on TEM images. (**a**) MS1; (**b**) MS2; (**c**) MS3.

The morphology and size of the azoxystrobin microspheres were further characterized by TEM. As observed in Figure 5, the microspheres presented a uniform regular spherical shape. The dried mean particle sizes of MS1, MS2, and MS3 were 119.4 nm, 307.5 nm, and 2609.2 nm (see Figure 6), respectively, according to the statistics from the TEM images. The statistical particle sizes of MS1, MS2, and MS3 were 8.8%, 13.0%, and 15.2%, smaller than the values determined by DLS. This may illustrate the fact that TEM samples were displayed in a dried state of particles, resulting in the shrinkage of microspheres. In contrast, DLS samples were reflected on hydrodynamic sizes of particles, showing a single particle or aggregate sizes. The similar phenomenon had been reported in other research papers [39,40,41,42,43].

### 3.2. Azoxystrobin Loading and Encapsulation Efficiency 

**Figure 7:**
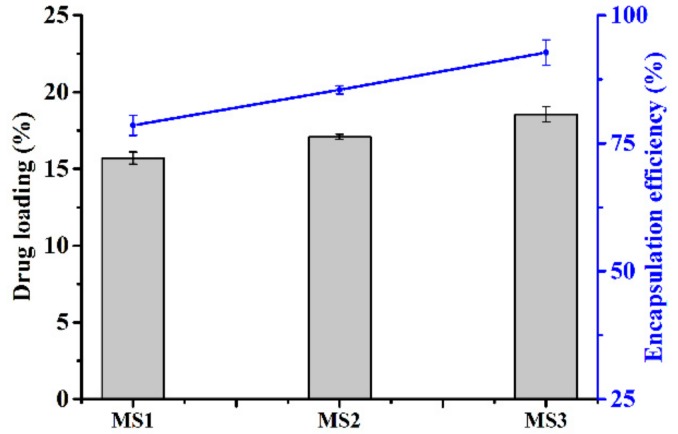
Effect of particle sizes on drug loading and encapsulation efficiency.

The effect of nanoparticle size on the azoxystrobin loading and encapsulation efficiency of microspheres is depicted in Figure 7. The drug loading of MS1, MS2, and MS3 was 15.7%, 17.1%, and 18.5%, respectively. The azoxystrobin encapsulation efficiency of MS1, MS2, and MS3 was 78.5%, 85.4%, and 92.7%, respectively. Because the hydrodynamic mean particle sizes were MS1 < MS2 < MS3, it was found that the drug loading and encapsulation efficiency showed a positive correlation with the particle size of microspheres. The possible reason was that the larger microspheres possessed more spatial hydrophobic structures to load the more insoluble pesticide. Additionally, the extra processing steps of smaller microsphere emulsification potentially had a negative effect on drug loading. As reported, the larger-sized mPEG-PLGA microspheres had a capacity of loading more prochloraz compared with smaller analogues [44].

### 3.3. In Vitro Release Profiles of Microspheres

**Figure 8:**
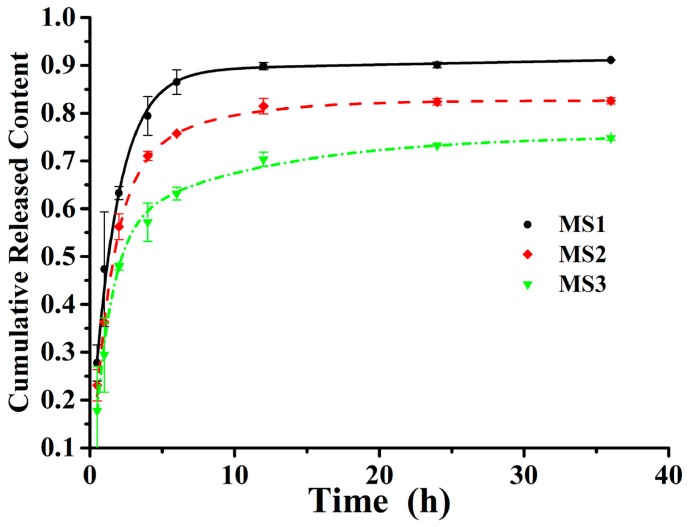
Cumulative release profiles of the azoxystrobin microspheres.

Figure 8 shows the accumulative release patterns of azoxystrobin from MS1, MS2, and MS3 in release medium containing 50% (*v/v*) MeOH for 1.5 d. In the initial 0.5 h, the percentages of released azoxystrobin from MS1, MS2, and MS3 were 27.75%, 23.09%, and 17.74%, respectively. After 24 h, the cumulative releases of azoxystrobin from MS1, MS2, and MS3 were 90.09%, 82.34%, and 73.23%, respectively. In addition, the release rate of azoxystrobin from PLA microspheres was dependent on the particle sizes. The drug loaded on microspheres mainly existed in two forms: one part of the active ingredient was dispersed inside microspheres; the other was adsorbed on the surface of microspheres [45,46]. Based on polymer carriers, the sustained drug release system was often intricate. However, it usually involved two major expulsion processes: first, an initial burst of release drug had been derived from the microspheres’ surface; and then, the slower continued release stage had been dependent on the diffusion and degradation of polymer carriers after the initial burst period [47]. The microsphere with the smallest particle size had the fastest release speed and the largest cumulative release percentage, consistent with the previous literature [44,48]. Currently, two exponential diphase kinetic functions may be the prevalent diffusion kinetics equation [49,50]. The azoxystrobin release behaviors from the microspheres corresponded well with the two exponential diphase kinetics fitting equations, with an R^2^ value of more than 0.99 (see Table 1). Therefore, two exponential diphase kinetic equations were able to illustrate the released mechanism of the azoxystrobin PLA microspheres’ in vitro test.

### 3.4. Stability of the Microspheres

**Figure 9:**
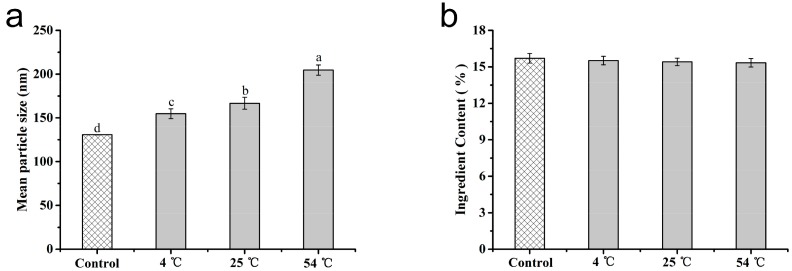
Storage stability of the MS1 microsphere for 14 d: (**a**) hydrated mean particle size; (**b**) active ingredient content. For hydrated mean particle sizes and ingredient contents, different lowercase letters indicated statistically significant differences (one-way ANOVA, Student–Newman–Keuls (SNK) test, *p* < 0.05).

As one vital assessment index of the stability of suspensions, the hydrodynamic mean particle size of azoxystrobin MS1 was monitored during storage. The variations of hydrated particle sizes were investigated by DLS after storage at 4 °C, 25 °C, and 54 °C for 14 d. As can be seen in Figure 9a, the initially mean size of MS1 was 130.9 ± 0.2 nm. After 14-d storage at 4 °C, 25 °C, and 54 °C, the sizes increased to 154.8 ± 5.6 nm, 166.8 ± 6.8 nm, and 204.7 ± 5.9 nm at 4 °C, 25 °C, and 54 °C, respectively. The growth proportions were 18.26%, 27.42%, and 56.36% for 4 °C, 25 °C, and 54 °C storage. The slight increase at 4 °C and 25 °C implied the desired physical stability of MS1 without remarkable particle agglomeration. Nonetheless, the hydrated particle size presented a moderate growth at 54 °C for 14 d. As is well known, the agglomeration of particles could be induced by high temperature [51,52]. Furthermore, the storage temperature of 54 °C approached the glass transition temperature of PLA [53,54,55]. As shown in Figure 9b, the initially active ingredient content of MS1 was 15.70%. After 14 d of storage evaluation, the active ingredient contents of MS1 decreased to 15.51% and 15.41% at 4 °C and 25 °C. An accelerated stability test was implemented according to the rapid evaluation standard CIPAC MT 46 and GB/T 19136-2003. The active ingredient content of MS1 was reduced to 15.33% at thermal storage (54 °C) for 14 d. The decomposition rate of azoxystrobin in MS1 was 2.37% in the accelerated test. All of the decomposition rates met the criteria of less than 5% recorded in CIPAC MT 46 and GB/T 19136-2003. These findings suggested that azoxystrobin-PLA MS1 maintained a good physical and chemical stability over the storage test.

### 3.5. Effect of Microsphere Size on Contact Angle

**Figure 10:**
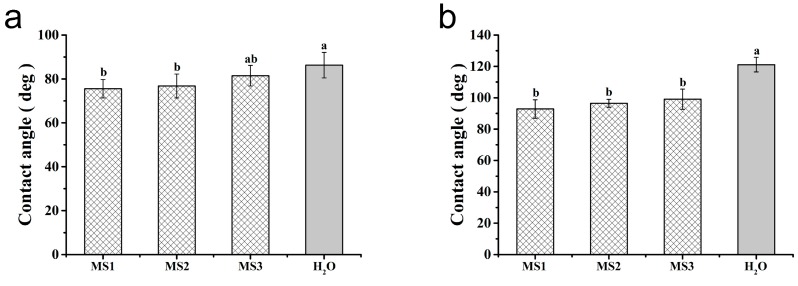
Contact angles of the azoxystrobin microspheres on the leaf surfaces: (**a**) cucumber leaf; (**b**) cabbage leaf. For contact angles on cucumber leaves and cabbage leaves, different lowercase letters indicated statistically significant differences (one-way ANOVA, SNK test, *p* < 0.05).

The adhesion of pesticides on target crops played a vital role in enhancing their effective availability [56,57,58]. The static contact angles were determined to estimate the pesticide wettability on cucumber and cabbage leaves. As described in Figure 10, the contact angles of water on the surfaces of the cucumber and cabbage leaves were 86° ± 6° and 121° ± 5°, respectively, implying the slightly hydrophilic properties of cucumber and the representative hydrophobic feature of cabbage. In comparison, the contact angles of the MS1, MS2, and MS3 on the cucumber leaves’ surface were 76° ± 4°, 77° ± 5°, and 81° ± 5° (see Figure 10a), respectively. Meanwhile, on the surface of the cabbage leaves, the contact angles of the MS1, MS2, and MS3 were 93° ± 6°, 97° ± 3°, and 99° ± 6° (see Figure 10b), respectively. With respect to the particle sizes of azoxystrobin microspheres, the contact angles had a positive correlation with the size on the surfaces of cucumber and cabbage. Based on the previous literature, the smaller particles held the smaller contact angles, due to their larger surface to volume ratio and surface free energy [59,60]. This result demonstrated that reducing particle size of azoxystrobin microspheres was able to improve the wettability on cucumber and cabbage leaves, further enhancing their adhesion.

### 3.6. In Vitro Antagonistic Activity of Microspheres

**Figure 11:**
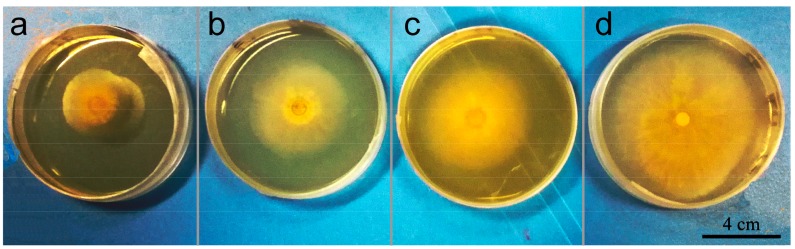
Antifungal efficacies of azoxystrobin microspheres against *Colletotrichum higginsianum* Sacc. (**a**) MS1; (**b**) MS2; (**c**) MS3; (**d**) CK.

Antagonistic activities of azoxystrobin microspheres were investigated in vitro against *Colletotrichum higginsianum* Sacc A 5-mm-diameter mycelial disk was inoculated onto the PDA plate, containing 10 μg/mL of MS1, MS2, or MS3 at 27 °C for two days. The inhibitory effects of the azoxystrobin microspheres on *Colletotrichum higginsianum* Sacc growth are revealed in Figure 11. The mycelial colony diameters of MS1, MS2, MS3, and CK were 27.0 mm, 44.5 mm, 49.5 mm, and 77.5 mm, respectively. The inhibitory rates of MS1, MS2, and MS3 were 69.66%, 45.52%, and 38.62%, respectively. As can be seen from Table 2, the 50% lethal concentration (LC_50_) values of MS1, MS2, and MS3 were 2.0386 μg/mL, 12.7246 μg/mL, and 21.2905 μg/mL against *Colletotrichum higginsianum* Sacc, respectively. The lowest LC_50_ value of the 130.9 nm azoxystrobin MS1 indicated that its toxicity index was over 10-fold that of the azoxystrobin MS3 with 3078.0 nm size. The results showed that the microspheres with smaller particle size produced the better antagonistic effects in the in vitro test. 

### 3.7. Effect of Microsphere Size on Intracellular Uptake

**Figure 12:**
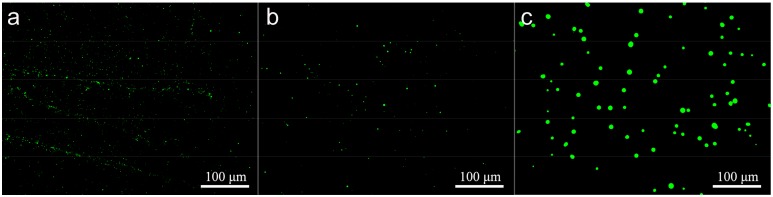
Fluorescence microscopy images of coumarin 6-loaded azoxystrobin microspheres. (**a**) MS1; (**b**) MS2; (**c**) MS3.

**Figure 13:**
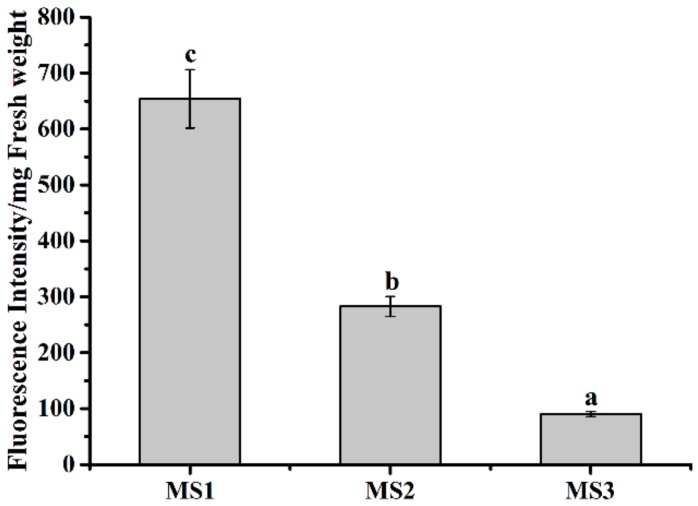
In vivo uptake efficiencies of coumarin 6-loaded azoxystrobin microspheres against *Colletotrichum higginsianum* Sacc. For coumarin 6 fluorescent values, different lowercase letters indicated statistically significant differences (one-way ANOVA, SNK test, *p* < 0.05).

To evaluate the mycelial intracellular uptake efficiency of MS1, MS2, and MS3, the fluorescent coumarin 6-loaded azoxystrobin microspheres were fabricated and incubated with *Colletotrichum higginsianum* Sacc. The fluorescent microscopy images of the coumarin 6-loaded azoxystrobin microspheres were displayed in Figure 12. The results indicated that coumarin 6-loaded azoxystrobin microspheres were spherical shape, without free fluorescence. In order to illustrate the mycelial intracellular uptake relationship with particle sizes of microspheres, relative fluorescent quantification of coumarin 6 was measured using a spectrofluorometer (see Figure 13). Compared to the mycelial intracellular fluorescence value of coumarin 6-loaded MS3, significant increases of fluorescence were determined in coumarin 6-loaded MS1 and MS2 treatment groups. In comparison, the coumarin 6 fluorescent values of MS1 and MS2 were approximately 7.25 and 3.13 times that of MS3, respectively. Those findings implied that the mycelial intracellular uptake efficiency was inversely correlated with the particle size of azoxystrobin microspheres. Both the particle size and surface chemical modification of nanoparticles were decisive factors that affected their biological efficiency and application [61,62]. In a previous study, Raza et al. [63] described the smaller silver nanoparticles as having greater antibacterial efficacy than the larger ones against *P. aeruginosa* and *E. coli*, for the higher surface/volume ratio and faster release rate. The dissolution rate of active ingredient and the transmembrane permeability of particles were size dependent [22,64,65]. Therefore, the remarkably enhanced antagonistic effect of 130.9 nm azoxystrobin microsphere could be attributed to the improved dissolution rate and transmembrane permeability of small particles.

### 3.8. Effect of Microsphere Size on Intracellular ROS

**Figure 14:**
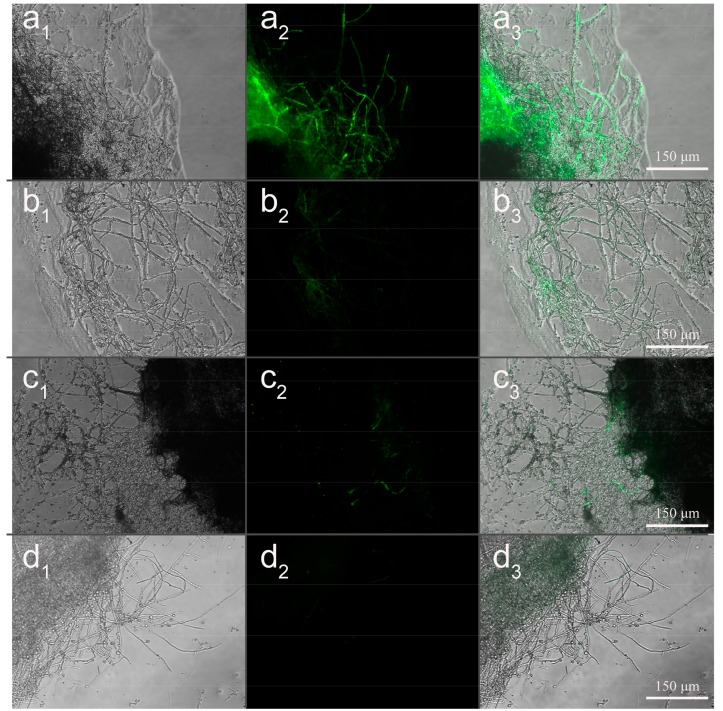
Fluorescence microscopy images of *Colletotrichum higginsianum* Sacc after treatment with the azoxystrobin microspheres. (**a**) MS1; (**b**) MS2; (**c**) MS3; (**d**) CK. Note: 1, bright field; 2, dark field; 3, overlay.

**Figure 15:**
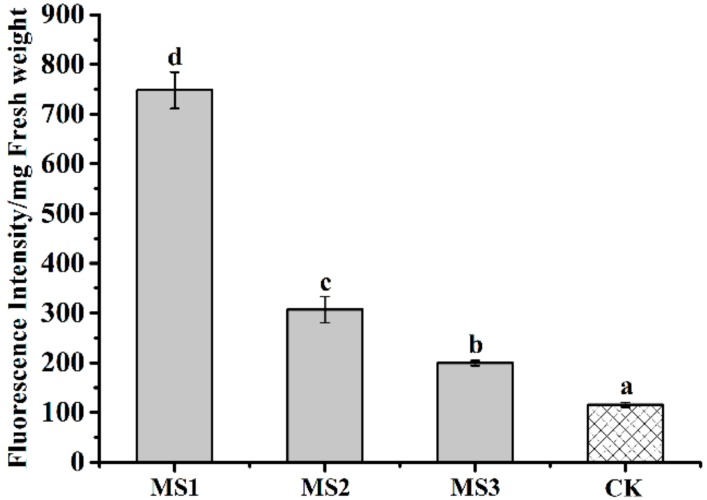
Effects of the azoxystrobin microspheres on intracellular ROS in *Colletotrichum higginsianum* Sacc. For fluorescent values, different lowercase letters indicated statistically significant differences (one-way ANOVA, SNK test, *p* < 0.05).

**Figure 16:**
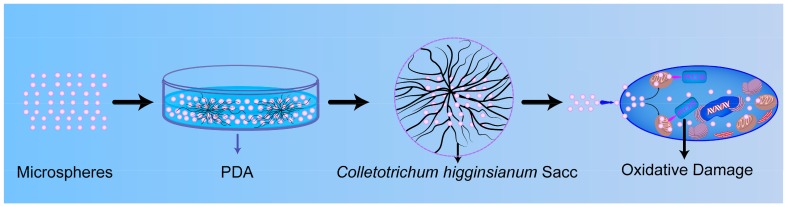
Antifungal schematic illustration of the azoxystrobin microspheres against *Colletotrichum higginsianum* Sacc.

To estimate the effect of MS1, MS2, and MS3 on mycelia oxidative stress, intracellular ROS of *Colletotrichum higginsianum* Sacc were tested by DCFH-DA assay. The dichlorofluorescein (DCF) fluorescent microscopy images of *Colletotrichum higginsianum* Sacc were shown in Figure 14. During the normal physiological metabolism of mycelia, reactive oxygen species were generated. The slight fluorescence was observed in the CK group (see Figure 14d). Compared with the CK group, the distribution and intensity of the DCF fluorescent signal were enhanced in the treatments with the azoxystrobin microspheres, presenting a particle size-dependent effect. In order to illuminate the relationship between microsphere particle sizes and the fluorescent signal intensity generated by ROS, relative quantification of DCF was conducted using a spectrofluorometer (see Figure 15). Compared to the fluorescence value of CK mycelia, significant increases of fluorescence were examined in the azoxystrobin microsphere treatment groups. In comparison, the DCF fluorescent values of MS1 and MS2 were approximately 6.27 and 2.57 times that of MS3 against *Colletotrichum higginsianum* Sacc. This suggested that the level of oxidative damage was negatively correlated with the particle size of the microspheres. Due to superior transmembrane permeability, the active ingredient with smaller particle size was more accessible to the target site [66]. The antifungal mechanism of azoxystrobin microspheres is depicted in Figure 16. As an inhibitor of mitochondrial electron chain transmission, azoxystrobin interdicted the synthesis of ATP by binding to the ubiquinol oxidizing (Qo) site of the complex III, which was located in the inner mitochondrial membrane [67,68,69]. This inhibition caused the electron to escape from the mitochondrial electron transport chain, which triggered cellular oxidative stress by generating excess ROS, even damaging cell membrane [70,71]. When *Fusarium graminearum* and *Microdochium nivale* were treated with azoxystrobin (100 μg/mL), the values of fluorescence from mycelia were greater than that of the treatment with dimethyl sulfoxide as the control group, suggesting that azoxystrobin increased the generation of ROS [72]. According to Table 2 and Figure 15, the antagonistic activities of the azoxystrobin microspheres were consistent with the extent of their oxidative damage. Therefore, the oxidative damage induced by ROS was an important signal pathway to inhibit fungi by azoxystrobin.

### 3.9. Effect of Microsphere Size on Antioxidase Activities

**Figure 17:**
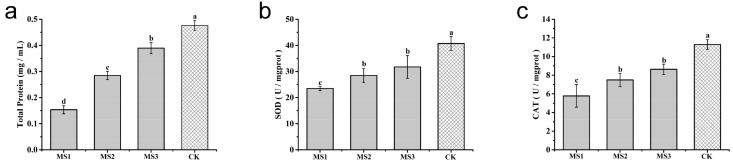
Effect of the azoxystrobin microspheres on soluble protein contents and anti-oxidase activities against *Colletotrichum higginsianum* Sacc. (**a**) Total protein; (**b**) SOD; (**c**) CAT. For soluble protein contents and anti-oxidase activities, different lowercase letters indicated statistically significant differences (one-way ANOVA, SNK test, *p* < 0.05).

To illustrate the mechanism of the azoxystrobin microspheres with controllable particle sizes in inhibiting *Colletotrichum higginsianum* Sacc growth, antioxidase activities were measured to reveal the oxidative damage of mycelia after different treatments. The soluble protein contents of *Colletotrichum higginsianum* Sacc exposed to azoxystrobin microspheres are presented in Figure 17a. Compared to the CK group, a significant decrease of the soluble protein content was analyzed in the azoxystrobin-treated groups over two days. This demonstrated that the expression of soluble protein was inhibited and an oxidative damage of mycelia was potentially generated. In the long term of evolution, series of antioxidant defense systems have developed in organisms, including SOD and CAT [73,74]. Converting superoxide anios to H_2_O_2_ was mainly catalyzed by SOD. The change from H_2_O_2_ to H_2_O principally depended on CAT [75,76]. Therefore, SOD and CAT were considered the foremost antioxidant enzymes for protecting organisms from oxidative damage. The changes of SOD activities in *Colletotrichum higginsianum* Sacc are displayed in Figure 17b. Compared to the CK group, the SOD activities were prominently inhibited in the azoxystrobin-treated groups. The smaller azoxystrobin microspheres exhibited lower activities of SOD. A similar experimental result emerged in the measurement of CAT activities (see Figure 17c). The lower activities of SOD and CAT indicated that the antioxidant defense system of *Colletotrichum higginsianum* Sacc, to some extent, was likely to be damaged. Consistent with Figure 15 and Figure 17, the smaller azoxystrobin microsphere was capable of leading to more seriously oxidative damage against *Colletotrichum higginsianum* Sacc.

## 4. Conclusions

To illustrate the particle size effect of azoxystrobin microspheres on their antagonistic efficacy against *Colletotrichum higginsianum* Sacc, the size-controlled azoxystrobin microspheres were fabricated by an *o/w* solvent evaporation method. The azoxystrobin microspheres exhibited an approximate uniform size distribution and regular spherical shape. The drug loading and encapsulation efficiency of microspheres were proportional to their particle sizes. The release kinetic profiles were firmly consistent with two exponential diphase kinetic equations, following an R^2^ value over 0.99. Additionally, the smaller microspheres presented faster release speed and larger cumulative percentage. Due to the larger surface/volume ratio and surface free energy, the particle size reduction of microspheres was able to enhance the adhesion to the cucumber and cabbage leaves. Attributed to a faster dissolution rate of active ingredient and a superior transmembrane permeability, the smaller azoxystrobin microsphere exhibited stronger antagonistic activity and lower activities of SOD and CAT against *Colletotrichum higginsianum* Sacc. The oxidative damage induced by ROS was an important signal pathway for the inhibition of fungi by azoxystrobin. In conclusion, the extent of oxidative damage had a particle size-dependent effect. Therefore, particle size would be regarded as a crucial index for improving the efficiency of fungicide delivery systems.

## Figures and Tables

**Table 1:** Fitting equations of the release profiles of the azoxystrobin microspheres.

Sample	Kinetic Equations	*R* ^2^
MS1	y = 796.50 − 0.81e^−0.57x^ − 795.61e^−0.75x^	0.9983
MS2	y = 0.83 − 0.67e^−0.74x^ − 0.17e^−0.17x^	0.9999
MS3	y = 0.75 − 0.59e^−0.85x^ − 0.20e^−0.09x^	0.9971

**Table 2:** Indoor toxicity of azoxystrobin microspheres against *Colletotrichum higginsianum* Sacc.

Sample	Toxicity Regressive Equation	*R* ^2^	LC_50_ (μg/mL)	Toxicity Index
MS1	*y* = 0.4106 + 0.2961 *x*	0.9995	2.0386	10.1137
MS2	*y* = 0.2445 + 0.2149 *x*	0.9913	12.7246	1.6732
MS3	*y* = 0.1451 + 0.2118 *x*	0.9620	21.2905	1
